# CXCR3^+^ T Follicular Helper Cells Induced by Co-Administration of RTS,S/AS01B and Viral-Vectored Vaccines Are Associated With Reduced Immunogenicity and Efficacy Against Malaria

**DOI:** 10.3389/fimmu.2018.01660

**Published:** 2018-07-25

**Authors:** Georgina Bowyer, Amy Grobbelaar, Tommy Rampling, Navin Venkatraman, Danielle Morelle, Ripley W. Ballou, Adrian V. S. Hill, Katie J. Ewer

**Affiliations:** ^1^The Jenner Institute, University of Oxford, Oxford, United Kingdom; ^2^GSK Vaccines, Rixensart, Belgium

**Keywords:** malaria, vaccine, T follicular helper cell, antibody, efficacy, CXCR3, RTSS viral-vectored vaccines

## Abstract

A malaria vaccine strategy targeting multiple lifecycle stages may be required to achieve a high level of efficacy. In two Phase IIa clinical trials, we tested immunogenicity and efficacy of RTS,S/AS01B administered alone, in a staggered regimen with viral-vectored vaccines or co-administered with viral-vectored vaccines. RTS,S/AS01B induces high titers of antibody against sporozoites and viral-vectored vaccines ChAd63 ME-TRAP and MVA ME-TRAP induce potent T cell responses against infected hepatocytes. By combining these two strategies, we aimed to improve efficacy by inducing immune responses targeting multiple parasite antigens. Vaccination with RTS,S/AS01B alone or in a staggered regimen with viral vectors produced strong immune responses and demonstrated high levels of protection against controlled human malaria infection. However, concomitant administration of these vaccines significantly reduced humoral immunogenicity and protective efficacy. Strong Th1-biased cytokine responses induced by MVA ME-TRAP were associated with a skew in circulating T follicular helper cells toward a CXCR3^+^ phenotype and a reduction in antibody quantity and quality. This study illustrates that while a multistage-targeting vaccine strategy could provide high-level efficacy, the regimen design will require careful optimization.

## Introduction

Despite years of remarkable success in reducing malaria morbidity and mortality, progress appears to have stalled with 216 million new cases in 2016, 5 million more than 2015 (1). An efficacious vaccine could be an essential tool to enable any further reduction in morbidity and mortality, and for the ultimate goal of eradication (2). The most advanced vaccine candidate, RTS,S, has shown significant short-term protective efficacy and has completed testing in a large Phase III trial (3–7). However, there remains a need to improve efficacy to achieve the goals laid out in the Malaria Vaccine Technology Roadmap (8). To improve efficacy, it may be necessary to develop a vaccine regimen targeting multiple stages of the parasite lifecycle (9). In addition to RTS,S, which targets the pre-liver stage, vaccines are being developed to target liver- and blood-stage parasites, or block parasite transmission and these could be combined into a multistage malaria vaccine program (9–12). It is likely that for a multistage vaccine regimen to provide high-level efficacy, it will need to induce both potent T cell and antibody responses (9).

The primary mechanism by which RTS,S induces protection appears to be antibody responses against the NANP repeat region of the circumsporozoite protein (CSP) on the sporozoite surface (13–16). The viral-vectored vaccines used in this study were chimpanzee adenovirus serotype 63 (ChAd63) and modified vaccinia virus Ankara (MVA), both expressing a multi-epitope (ME) string fused to the *Plasmodium falciparum* protein thrombospondin-related adhesive protein (TRAP). The ME string contains 17 epitopes from potentially protective *P. falciparum* and bacille Calmette–Guérin antigens in addition to epitopes from tetanus toxoid. TRAP is expressed on the surface of sporozoites and contains a thrombospondin domain that binds to heparin sulfate proteoglycans to facilitate sporozoite entry into host hepatocytes. ChAd63 ME-TRAP and MVA ME-TRAP provide protection by inducing CD8^+^ T cell responses against infected hepatocytes (17). When RTS,S/AS01B and viral-vectored vaccines were tested in a combination regimen, efficacy against controlled human malaria infection (CHMI) was higher for volunteers receiving the combination of vaccines (14/17 subjects protected; vaccine efficacy (VE) 82.4% [95% CI 64–100]) than for those receiving RTS,S/AS01B alone (12/16 subjects protected; VE 75% [95% CI 54–96]), suggesting that TRAP-specific T cell responses could add to the protective effect of RTS,S-induced antibody responses (18). In that study, NANP IgG titers in the combination group were comparable to those in the group given RTS,S/AS01B alone and were significantly higher in protected individuals. Titers of IgG against NANP were negatively correlated with parasitemia at day 7.5, indicating a reduced liver to blood inoculum. However, as viral-vectored vaccinations were given at staggered time points, a minimum of 2 weeks after RTS,S/AS01B, this regimen required five separate clinic visits over a period of 10 weeks. For a vaccine regimen to be logistically and economically feasible for deployment in malaria-endemic regions, the number of clinic visits should be reduced. For this reason, we conducted a further Phase I/IIa clinical trial to assess concomitant administration of RTS,S/AS01B with viral-vectored vaccines (Rampling et al. manuscript under review). On the basis of high efficacy in two previous trials, additional groups were included to test a reduced third dose of RTS,S/AS01B (1/5th, 10 μg) (14, 19). In this study, co-administration of these two vaccine platforms resulted in a significant reduction in humoral immunogenicity and efficacy with only 11/19 volunteers protected (VE 57.9% 95% CI [33.2–76.3]), compared with 14/17 (82.4% 95% CI [54.7–93.9]) in the group receiving RTS,S/AS01B alone.

Durable, high-affinity IgG is generated in germinal center (GC) reactions in secondary lymphoid organs, during which B cells undergo class-switching, somatic hypermutation, and differentiation into memory B cells and plasma cells. T follicular helper cells (Tfh) expressing CD4 and CXCR5 and the transcription factor Bcl-6 provide essential help to B cells for this process in the form of cytokine production (IL-21) and the expression of costimulatory molecules (CD40L, ICOS) (20, 21). Circulating PD1^+^CXCR5^+^CD45RA^−^ CD4^+^ T cells appear to be a peripheral counterpart of conventional lymphoid resident Tfh, may represent GC responses, and are a useful tool for clinical trials in which lymphoid tissue is rarely available for analysis (22–24). Circulating T follicular helper cells (cTfh) can be further defined by differential expression of chemokine receptors CXCR3 and CCR6: Th17-like (cTfh17) CXCR3^−^CCR6^+^, double-positive CXCR3^+^CCR6^+^, Th1-like (cTfh1) CXCR3^+^CCR6^−^, and Th2-like (cTfh2) CXCR3^−^CCR6^−^. These subsets have been associated with varying degrees of helper activity in different contexts (25). In particular, CXCR3^−^ Tfh have been associated with the production of broadly neutralizing antibodies against HIV and Tfh17 induced by rVSV-ZEBOV vaccination were associated with antibody responses against Ebola (26, 27). Therefore, the type of cTfh induced by vaccination may be an indicator of the quality of the GC reaction and the resulting antibodies produced.

To investigate the mechanisms underlying the reduction in humoral immunogenicity after co-administration of ChAd63-MVA ME-TRAP and RTS,S, we conducted a thorough analysis of the differences in antibody quality and cTfh responses in volunteers receiving RTS,S/AS01B alone (R), RTS,S/AS01B given with viral vectors in a staggered regimen (R2V) or co-administered (R + V). Trial regimens are summarized in Table 1. This is the first study to assess the impact of vaccine co-administration on the cTfh response in humans and also defines a functional antibody quality that may explain the improved efficacy observed in RTS,S regimens with a reduced third dose.

**Table 1:** Vaccination schedules.

		Co-administration study (VAC59)					Staggered study (VAC55)		
		RTS,S/AS01B (R)		RTS,S/AS01B co-administered with viral vectors (R + V)			RTS,S/AS01B and viral vectors staggered (R2V)	RTS,S/AS01B (R)	
Group	G1 R-R-R	G2 R-R-r	G3 RA-RM-RM	G4 RA-RM-rM	Controls	R-A-R-R-M	R-R-R	Controls
No. volunteers enrolled	10	10	10	11	4	20	17	6
No. volunteers at C-1	9	10	10	9	4	17	16	6
No. volunteers challenged	8	9	10	9	4	17	16	6
Week 0	R	R	RA	RA		R	R	
Week 2						A		
Week 4	R	R	RM	RM		R	R	
Week 8	R	r	RM	rM		R	R	
Week 10						M		
Week 11	CHMI	CHMI	CHMI	CHMI	CHMI			
Week 12						CHMI	CHMI	CHMI
Efficacy: sterilely protected volunteers	6/8 (75%)	8/9 (89%)	6/10 (60%)	5/9 (56%)	0/4	(14/17) 82%	(12/16) 75%	0/6

*R, 50 µg RTS,S/AS01B; r, 10 µg RTS,S/AS01B; A, 5 × 10^10^ viral particles (vp) ChAd63 ME-TRAP; M, 2 × 10^8^ plaque-forming units (pfu) MVA ME-TRAP; CHMI, controlled human malaria infection; (R), RTS,S/AS01B groups; (R2V), RTS,S/AS01B and viral-vectored vaccinations staggered by 2 weeks; (R + V), RTS,S/AS01B and viral vectors co-administered*.

## Materials and Methods

### Samples and Study Details

Full details of these studies are available in the clinical trial reports [(18), Rampling et al. manuscript under review]. Healthy adult volunteers were recruited and vaccinated at four UK sites, in Oxford, Southampton, London, and Surrey. The CHMI procedure was performed as previously described using five infectious bites from *P. falciparum* 3D7-strain infected *Anopheles stephensi* mosquitoes at Imperial College, London (28). All subjects were infected with a single batch of infected mosquitoes for each trial, supplied by the Department of Entomology, Walter Reed Army Institute of Research, Washington DC, USA. All vaccinations were administered intramuscularly into the deltoid region of the arm. For participants who received concomitant vaccinations, RTS,S/AS01B was administered first followed by the viral-vectored vaccine in the same site no longer than 5 min later.

### Ethics Statement

All volunteers gave written informed consent prior to participation, and the studies were conducted according to the principles of the Declaration of Helsinki and in accordance with Good Clinical Practice. The trials were registered with ClinicalTrials.gov (Ref: NCT01883609 and NCT02252640). The study protocols were approved by the UK National Research Ethics Service, Committee South Central—Oxford A (Refs: 13/SC/0208 and 14/SC/0227), the Western Institution Review Board (Ref: 20130698), and the UK Medicines and Healthcare products Regulatory Agency (Refs: 21584/0317/001-0001 and 21584/0333/001-0001). The Local Safety Committee provided safety oversight for both trials and GCP compliance was monitored by the Clinical Trials and Research Governance Team (CTRG) of the University of Oxford.

### Total IgG ELISA

ELISA 96-well plates were coated with a synthetic peptide (Eurogentec, Liège, Belgium) based on the PfCSP repeat region ((NANP)_6_C) diluted to 0.2 µg/mL in 100 µL dPBS per well and incubated overnight at room temperature (RT). Plates were washed six times with PBS containing 0.5% Tween-20 (PBS/T) and blocked with casein for 1 h at RT. Plates were washed again and serum samples diluted in casein at 1:100, 1:500, 1:1,000, or 1:5,000, were added for 2 h at RT. After washing again, secondary antibody (goat anti-human IgG conjugated to alkaline phosphatase, Sigma) was added at 1:1,000 in casein for 1 h at RT. Plates were washed a final time and developed using 4-nitrophenyl phosphate in diethanolamine buffer (Pierce, Rockford, IL, USA). Optical density (OD) was read at 405 nm using an ELx800 microplate reader (Bio-Rad, Hercules, CA, USA). A reference pool of positive serum formed a standard curve on each plate and was used to calculate ELISA units for each sample. An internal control was included on each plate to standardize between assays. All samples were tested in triplicate.

### Isotype ELISA

Isotype ELISAs were conducted as described above, except that all serum samples were diluted to 1:100 and added to the plate in duplicate wells on each of six plates. One of six secondary antibodies against IgG1, IgG2, IgG3, IgG4, IgM, or IgA was added to each plate at 1:1,000 in casein before developing as above. Blank wells and internal development controls were included on each plate. A “seropositive cut-off” value was calculated for each isotype or subclass using the mean plus 3 SDs of 36 UK malaria-naïve serum samples.

### Indirect Immunofluorescence Assay (IFA)

Chambered microscope slides coated with *P. falciparum* sporozoites were stored at −80°C until use. Slides were brought to RT and then fixed for 15 min in 4% paraformaldehyde. After washing twice in PBS for 5 min, slides were blocked for 1 h in casein. Slides were washed as before and 10 µL of serum sample diluted 1:100 in casein was added to each well. Slides were incubated for 30 min at RT in a humidity chamber then wells were individually washed with PBS three times for 5 min. Secondary antibody (anti-IgG-AlexaFluor488) was diluted 1:800 in casein and 15 µL was added to each well for 30–45 min in a humidity chamber at RT protected from the light. Slides were washed a final time, rinsed in distilled water and left to dry before mounting with DAPI-containing media. Slides were left to set overnight at 4°C before being examined under a Leica DMI3000 B microscope. Images were captured in QCapturePro software using brightfield illumination, GFP and DAPI filters at set exposure levels. ImageJ software was used to measure the median fluorescence intensity (MFI) for five sporozoites in each well and an average was taken.

### Inhibition of Sporozoite Invasion (ISI) Assay

The ISI assay was carried out as previously described (29). Human hepatoma cells (HC04) cultured in R10 medium (RPMI 1640 with 10% FCS, 1% penicillin/streptomycin, and 1% l-glutamine) were added to 96-well culture plates at 30,000 cells/well and left to settle overnight at 37°C, 5% CO_2_. Viable GFP-labeled *Plasmodium berghei* sporozoites expressing *P. falciparum* CSP at the *P. berghei* CSP locus (*P. berghei* PfCSP@CSP) were obtained by dissecting infected *A. stephensi* mosquitoes. Salivary glands were pooled into RPMI 1640 medium and homogenized. Sporozoites were counted and diluted to 100,000/mL in RPMI 1640. Culture medium was aspirated from the hepatoma cells then 100 µL of serum diluted 1:5 in R10 and 100 µL of sporozoite dilution (10,000 sporozoites, 10% final serum concentration) were added to each well. Samples were tested in duplicate and an average calculated. “Hepatoma only” wells and infectivity control wells that contained hepatoma cells and sporozoites but no serum were included. Pre-vaccination and C-1 samples were run for each volunteer. After incubation for 20–26 h at 37°C, medium was aspirated, and plates were washed with 90 µL/well dPBS. Cells were trypsinized, re-suspended in 65 µL dPBS with 1% bovine serum albumin (BSA), and acquired immediately using a BD LSRII. DAPI stain was added to each sample just before acquisition. Data were analyzed in Flow Jo software v10.6 (Tree Star Inc., Ashland, OR, USA) according to a predefined gating strategy. The percentage of sporozoite inhibition was calculated for each sample (average of duplicate wells) based on the reduction in the percentage of infected cells compared with the infectivity controls (average of 4–6 wells).

### *Ex Vivo* IFNγ ELISpot

*Ex vivo* ELISpot assays were performed for TRAP-specific T cell responses as previously described (10). Average responses were taken across triplicate wells, background subtracted and then responses in individual pools were summed.

### cTfh Phenotyping and ICS

Surface phenotyping of cTfh was carried out using cryopreserved peripheral blood mononucleocytes (PBMCs). Thawing was performed rapidly in a water bath and cells were rested for 2 h at 37°C with 5% CO_2_ and Benzonase at 25U/10^6^ PBMC (Novagen, Madison, WI, USA) before staining. For surface phenotyping 1–2 million PBMC were stained. Cells were washed in FACS buffer (PBS containing 0.1% BSA and 0.01% sodium azide) and stained with LIVE/DEAD aqua amine reactive dye (Life Technologies Ltd., Carlsbad, CA, USA) for 20 min at RT in the dark. Cells were washed in FACS buffer and a cocktail of antibodies for Tfh surface staining (Table 2) was added for 30 min at RT. Cells were washed again in FACS Buffer and re-suspended in PBS containing 1% paraformaldehyde, prior to acquisition on a BD LSR II using FACSDiva v6.2 (BD Biosciences, Franklin Lakes, NJ, USA) on the day of staining. Compensation control beads (OneComp Beads, eBioscience, San Diego, CA, USA, ArC Amine Reactive Beads, Invitrogen, Carlsbad, CA, USA) were stained according to the manufacturer’s instructions for compensation between parameters. A median of 100,000 live CD4^+^ cells were acquired [inter-quartile range (IQR) 25% = 68,406, 75% = 143,000] per sample. Data analysis was performed using Flow Jo v9.6.2 (Tree Star Inc.). IFNγ production was measured after overnight stimulation with 2 µg/mL of a pool of 31 peptides spanning the CSP antigen (15mers overlapping by 11 amino acids, at 2 µg/mL, all volunteers) or 10 µg/mL superantigen *Staphylococcus* enterotoxin B (SEB). Brefeldin A and monensin were added at 10 µg/mL after 2 h. The staining protocol was the same as for cTfh phenotyping, except that after surface staining, cells were permeabilized with fix/perm buffer (BD biosciences) then stained intracellularly at RT for 30 min with IFNγ-FITC (1:250, eBioscience), washed and fixed in 1% paraformaldehyde. Acquisition and analysis was performed as for cTfh phenotyping.

**Table 2:** Circulating T follicular helper cell phenotyping panel.

Marker	Fluorophore	Supplier	Clone	Dilution	Volume (μL)
CD3	Alexa Fluor 700	eBioscience	UCHT1	1:33	1.5
CD4	APC-eFluor 780	eBioscience	SK3 (SK-3)	1:50	1
CD45RA	eFluor450	BioLegend	HI101	1:50	1
CXCR5	PerCP-eFluor 710	BioLegend	MU5UBEE	1:16	3
CXCR3	APC	BioLegend	1C6/CXCR3	1:16	3
CCR6	PE	BioLegend	G034E3	1:16	3
PD-1	BV650	BioLegend	EH12.2H7	1:100	0.5
Live/dead	AmCyan	Invitrogen	N/A	1:500	0.1
IFNγ (in ICS assay)	FITC	eBioscience	4S.B3	1:250	0.2

### Multiplex Cytokine Assay

Between 1 and 2 million PBMC from the C-1 time point were plated per well in a 96-well plate and stimulated for 21 h at 37°C. Cells were stimulated either with a pool of 31 peptides spanning the CSP antigen (15mers overlapping by 11 amino acids, at 2 µg/mL, all volunteers) or 10^6^ pfu of MVA. Supernatants were taken and stored at −20°C in 96-well U-bottom polypropylene plates until use. Cytokine concentrations in the supernatants were measured using the LEGENDplex human Th cytokine panel 13-plex assay (BioLegend, San Diego, CA, USA) according to the manufacturer’s instructions. The samples were read on the same day on a BD LSR II using FACSDiva v6.2 (BD Biosciences) with 5,000 beads acquired per sample. Data analysis was conducted using the LEGENDplex data analysis software.

### Statistical Analysis

Data tested negative for a normal distribution by D’Agostina–Pearson omnibus normality test; therefore, non-parametric tests were used and medians with IQRs are presented. Mann–Whitney analysis was used to compare differences between two groups. Kruskal–Wallis with Dunn’s multiple comparisons post-test was used to compare responses across multiple groups at a given time point. Wilcoxon matched-pairs analysis was used to compare responses at two time points within a group. Spearman’s rank was calculated for correlations. *P* < 0.05 was considered significant and all *P* values are two-tailed. Analyses were performed in GraphPad Prism, version 7.

## Results

### Reduced Quantity and Quality of Antibody Responses When RTS,S/AS01B Is Co-Administered With Viral-Vectored Vaccines

**Figure 1:**
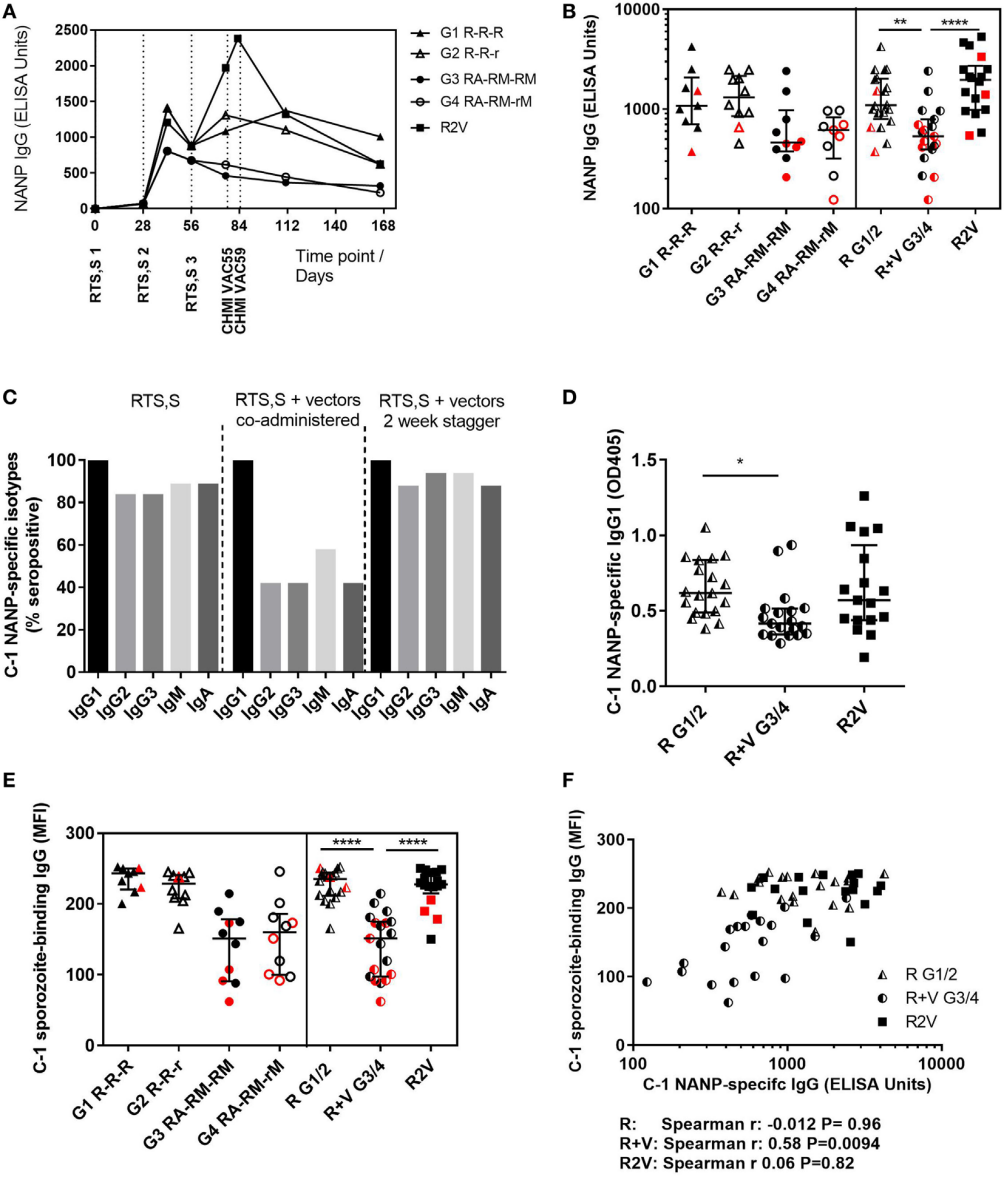
Antibody quantity and quality. **(A)** Median NANP-specific IgG time courses. **(B)** Total IgG titers against the CSP repeat region NANP in each group at D76 (2 weeks after third dose of RTS,S/AS01B), in G1/2 combined (R) and G3/4 combined (R + V) or in the staggered regimen (R2V), Kruskal–Wallis with Dunn’s post-test *P* < 0.0001. **(C)** NANP-specific isotype and IgG subclass responses at C-1. **(D)** NANP-specific IgG1 at C-1, Kruskal–Wallis *P* = 0.0062. **(E)** MFI of sporozoite-binding IgG at C-1 measured by IFA, Kruskal–Wallis *P* < 0.0001. **(F)** Relationship between NANP IgG titer and level of sporozoite-binding at C-1. Volunteers receiving RTS,S/AS01B alone (G1/2, R) Spearman *r*: −0.012, *P* = 0.96, RTS,S/AS01B co-administered with vectors (G3/4, R + V) Spearman *r*: 0.58, *P* = 0.0094 or RTS,S/AS01B and viral vectors given in a staggered regimen Spearman *r*: 0.06, *P* = 0.82. Medians + IQRs shown and non-protected volunteers highlighted in red for all column graphs. Abbreviations: R G1/2, RTS,S/AS01B vaccinated; R + V G3/4, RTS,S/AS01B and viral-vectored vaccines co-administered; R2V, RTS,S/AS01B and viral-vectored vaccines at a 2-week stagger; A, ChAd63 ME-TRAP; M, MVA ME-TRAP; R, 50 µg third dose of RTS,S/AS01B; r, 10 µg third dose of RTS,S/AS01B; CHMI, controlled human malaria infection; C-1, day before CHMI; CSP, circumsporozoite protein; TRAP, thrombospondin-related adhesive protein; IQRs, inter-quartile ranges; IFA, immunofluorescence assay; MFI, median fluorescence intensity.

Antibody responses against the NANP repeat region of CSP were measured in each of the clinical trials and reported separately (18) (Rampling et al. manuscript under review). Total NANP-specific IgG was measured by ELISA at baseline (day 0, D0), D28, D42, D56, D76 (the day before CHMI, C-1, in the co-administration trial) and 35 and 90 days after CHMI (C + 35, C + 90). In the staggered regimen trial, anti-NANP-specific IgG was also measured at D83 (C-1 for that trial) as the CHMI was 1 week later to accommodate the additional vaccinations (Figure 1A). Titers were comparable in all regimens after the first two vaccinations but failed to re-boost after the third vaccination in the co-administration regimen, resulting in significantly lower titers in these groups at D76 (Figure 1B, median ELISA units, R: 1,102 IQR [757–2,035], R + V: 533 IQR [394–790], R2V: 1,969 IQR [983–2,724] Kruskal–Wallis *P* < 0.0001). There were no significant differences in NANP IgG titers between groups receiving a full or reduced third dose of RTS,S/AS01B either alone or co-administered with viral-vectored vaccines. The highest titers were seen in the staggered regimen at D83 (4 weeks after the third dose of RTS,S/AS01B), although there was no comparable time point in the co-administration study.

Isotype and subclass responses were measured by ELISA against the NANP repeat region at C-1 in both trials (Figure 1C). Titers were measured for IgG1–4, IgM, and IgA. No NANP-specific IgG4 was detected in any volunteers (data not shown). Over 80% of volunteers given RTS,S/AS01B alone were seropositive for NANP-specific IgG2, IgG3, IgM, and IgA. There were significant reductions in seroconversion for these isotypes/subclasses in groups that received concomitant viral-vectored vaccinations, but not in the staggered regimen. All volunteers were positive for NANP IgG1 and titers were comparable in the RTS,S/AS01B only groups (R) and the staggered administration group (R2V), but were significantly reduced in the co-administration regimen (R + V) (Figure 1D, median OD, R: 0.617 IQR [0.488–0.835], R + V: 0.415 [0.343–0.514], R2V: 0.570 [0.438–0.935] Kruskal–Wallis *P* = 0.0062). There were no significant differences in IgG1 titers between groups receiving full or reduced third doses of RTS,S/AS01B (G1 R-R-R vs G2 R-R-r and G3 RA-RM-RM vs G4 RA-RM-rM, data not shown).

Antibody binding to fixed whole sporozoites was measured for all volunteers at C-1 using an indirect IFA. Sporozoite-binding IgG was significantly lower in the co-administration regimen but the staggered regimen was comparable to RTS,S/AS01B alone (Figure 1E, MFI, R: 238 IQR [213–245] R + V: 151 [98–175], R2V: 228 [215–245] Kruskal–Wallis *P* < 0.0001). There were no differences between groups receiving full or reduced third doses of RTS,S/AS01B. Sporozoite-binding was significantly associated with NANP IgG titers in the co-administration groups (Figure 1F, Spearman *r*: 0.58, *P* = 0.0094). There was no association in the RTS,S/AS01B alone or staggered regimens (Spearman *r*: −0.012, *P* = 0.96 and *r*: 0.058, *P* = 0.83, respectively), in which sporozoite-binding was higher for a given NANP IgG titer than in the co-administration regimen.

### ISI Is Associated With Protection From CHMI

**Figure 2:**
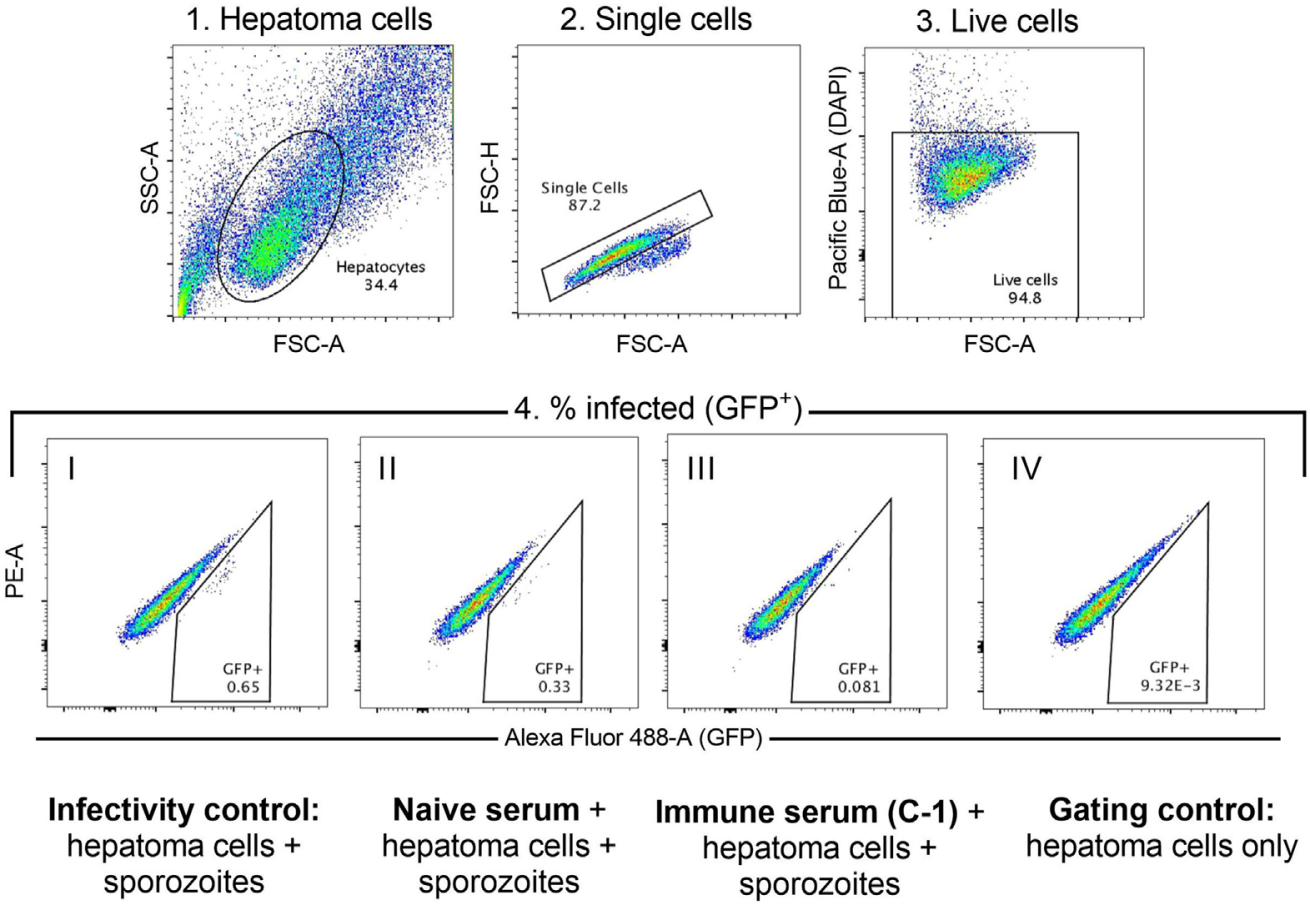
Inhibition of sporozoite invasion assay. Hierarchical gating strategy used to determine the percentage of hepatoma cells infected with GFP-expressing sporozoites. 1. Hepatoma cells are gated based on size to exclude debris and large cell clumps. 2. Singlets are gated to exclude smaller cell clumps. 3. Live cells (DAPI-negative) are gated to exclude dead cells. 4. Cells infected with the GFP-expressing sporozoites are GFP^+^. Cells are gated against both GFP (Alexa Fluor-AF488 channel, GFP^+^) and the adjacent channel (PE^−^) to exclude signal that is caused by autofluorescence. Representative populations in each of the conditions are shown: I. Infectivity control—sporozoite infection of hepatoma cells in the absence of any serum; II. Sporozoite infection of hepatoma cells in the presence of naïve serum (D0); III. Sporozoite infection of hepatoma cells in the presence of immune serum (C-1); IV. Gating control—hepatoma cells only, no sporozoites or serum added.

**Figure 3:**
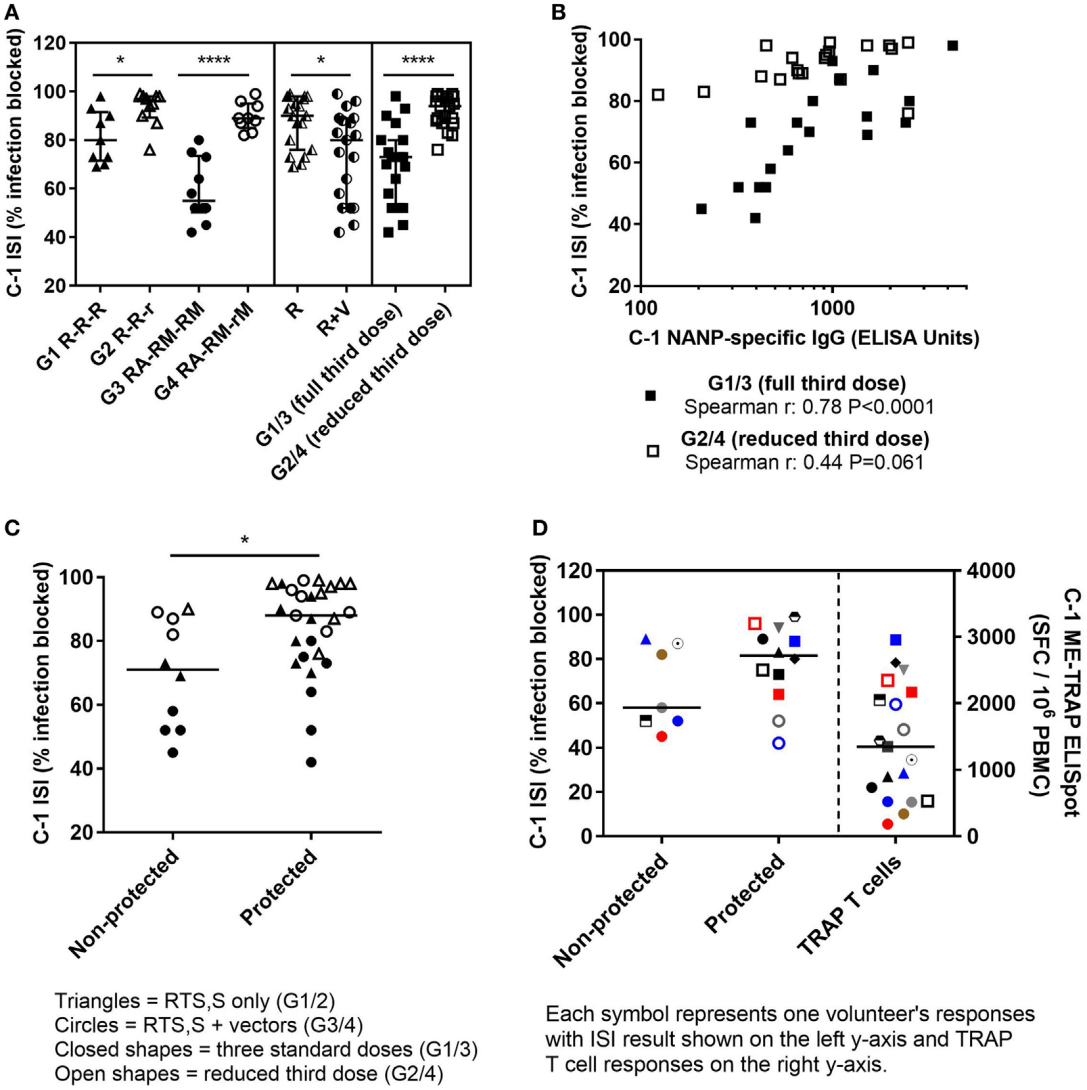
ISI results. **(A)** Percentage of sporozoite invasion into hepatoma cells that was blocked by serum at C-1. Comparison of groups given three standard doses of RTS,S/AS01B or a reduced (1/5th) third dose of RTS,S/AS01B. Mann–Whitney analyses G1 R-R-R vs G2 R-R-r *P* = 0.014, G3 RA-RM-RM vs G4 RA-RM-rM *P* < 0.0001, G1/2 (R) vs G3/4 (R + V) *P* = 0.016, G1/3 (full third dose of RTS,S/AS01B) vs G2/4 (reduced third dose of RTS,S/AS01B) *P* < 0.0001, medians + IQRs. **(B)** Relationship between C-1 NANP IgG titers and sporozoite-blocking ability in individuals who received three full doses of RTS,S/AS01B with or without vectored vaccines (G1 and G3, closed squares, Spearman *r*: 0.78, *P* < 0.0001) or a reduced third dose of RTS,S/AS01B with, or without vectored vaccines (G2 and G4, open squares, Spearman *r*: 0.44, *P* = 0.061). **(C)** Percentage of sporozoite invasion blocked by C-1 serum protected and non-protected individuals, Mann–Whitney *P* = 0.017. Closed triangles: G1, open triangles: G2, closed circles: G3, open circles: G4. **(D)** Percentage of sporozoite invasion blocked by serum at C-1 in protected and non-protected volunteers in G3/4, R + V (left *Y*-axis) and corresponding TRAP T cell responses (right *Y*-axis) at C-1 for each volunteer measured by IFNγ ELISpot, summed responses from T9/96 TRAP pools + ME, spot-forming cells per million PBMC (SFC/10^6^ PBMC), lines at medians. Abbreviations: R G1/2, RTS,S/AS01B vaccinated; R + V G3/4, RTS,S/AS01B and viral-vectored vaccines co-administered; R2V, RTS,S/AS01B and viral-vectored vaccines at a 2-week stagger; A, ChAd63 ME-TRAP; M, MVA ME-TRAP; R, 50 µg third dose of RTS,S/AS01B; r, 10 µg third dose of RTS,S/AS01B; CHMI, controlled human malaria infection; C-1: day before CHMI; ISI, inhibition of sporozoite invasion; PBMCs, peripheral blood mononucleocytes; TRAP, thrombospondin-related adhesive protein; IQRs, inter-quartile ranges.

The functional quality of vaccine-induced antibodies was assessed using an *in vitro* assay measuring the ability of serum to block sporozoite infection of hepatoma cells (29). A defined gating strategy was used to identify infected hepatoma cells (Figure 2). The percentage of infection blocked by vaccine-induced antibody was significantly lower in the co-administration regimen compared with RTS,S/AS01B alone (Figure 3A, median percentage infection blocked R, 90% IQR [76–98], R + V 80% [52–89], Mann–Whitney *P* = 0.016). However, blocking ability was significantly higher in groups receiving a reduced third dose of RTS,S/AS01B than those receiving three standard doses, even when this dose was co-administered with viral-vectored vaccines (Figure 3A, medians + IQRs G1 R-R-R: 80% [71.5–91.5], G2 R-R-r: 96% [89.3–98], G3 RA-RM-RM: 55% [50–73.5], G4 RA-RM-rM: 89% [85–95], Mann–Whitney analyses G1 R-R-R vs G2 R-R-r *P* = 0.014, G3 RA-RM-RM vs G4 RA-RM-rM *P* < 0.0001, G1 R-R-R and G3 RA-RM-RM vs G2 R-R-r and G4 RA-RM-rM *P* < 0.0001). ISI was significantly associated with C-1 NANP IgG titer for groups receiving three standard doses of RTS,S/AS01B (Figure 3B, G1 R-R-R and G3 RA-RM-RM Spearman *r*: 0.78, *P* < 0.0001) but not in groups receiving a reduced third dose, in which blocking ability was higher even at lower NANP titers (G2 R-R-r and G4 RA-RM-rM Spearman *r*: 0.44, *P* = 0.061). The relationship between blocking ability and the NANP-specific isotype/subclass titers was also assessed (Figure S1 in Supplementary Material). Relationships between all subclasses and isotypes tested and blocking ability demonstrated a similar pattern to total IgG, with a positive correlation in G1/3 and high blocking regardless of titer in G2/4. However, IgG1 was the only isotype that showed an association with blocking ability in the data set as a whole (G1–4, Spearman *r*: 0.42, *P* = 0.009).

Blocking ability was associated with protection from malaria after CHMI, with significantly higher percentages of infection blocked in protected than non-protected volunteers (Figure 3C, medians + IQRs protected: 88% [75–97], non-protected: 71% [52–87.5] Mann–Whitney *P* = 0.019). TRAP-specific T cell responses elicited by the viral-vectored vaccines were measured by IFNγ ELISpot and previously reported (Rampling et al. manuscript under review). T cell responses (IFNγ responses against summed T9/96 TRAP pools + the multi-epitope, ME) were not reduced by co-administration and responses were significantly higher in protected than non-protected individuals in these groups (Rampling et al. manuscript under review). Volunteers in the co-administration groups that were protected despite having low levels of sporozoite-blocking antibody had high TRAP-specific T cell responses (Figure 3D).

### Proportion of CXCR3^+^ cTfh Increases When RTS,S/AS01B Is Co-Administered With Viral-Vectored Vaccines and Negatively Correlates With Antibody Responses

**Figure 4:**
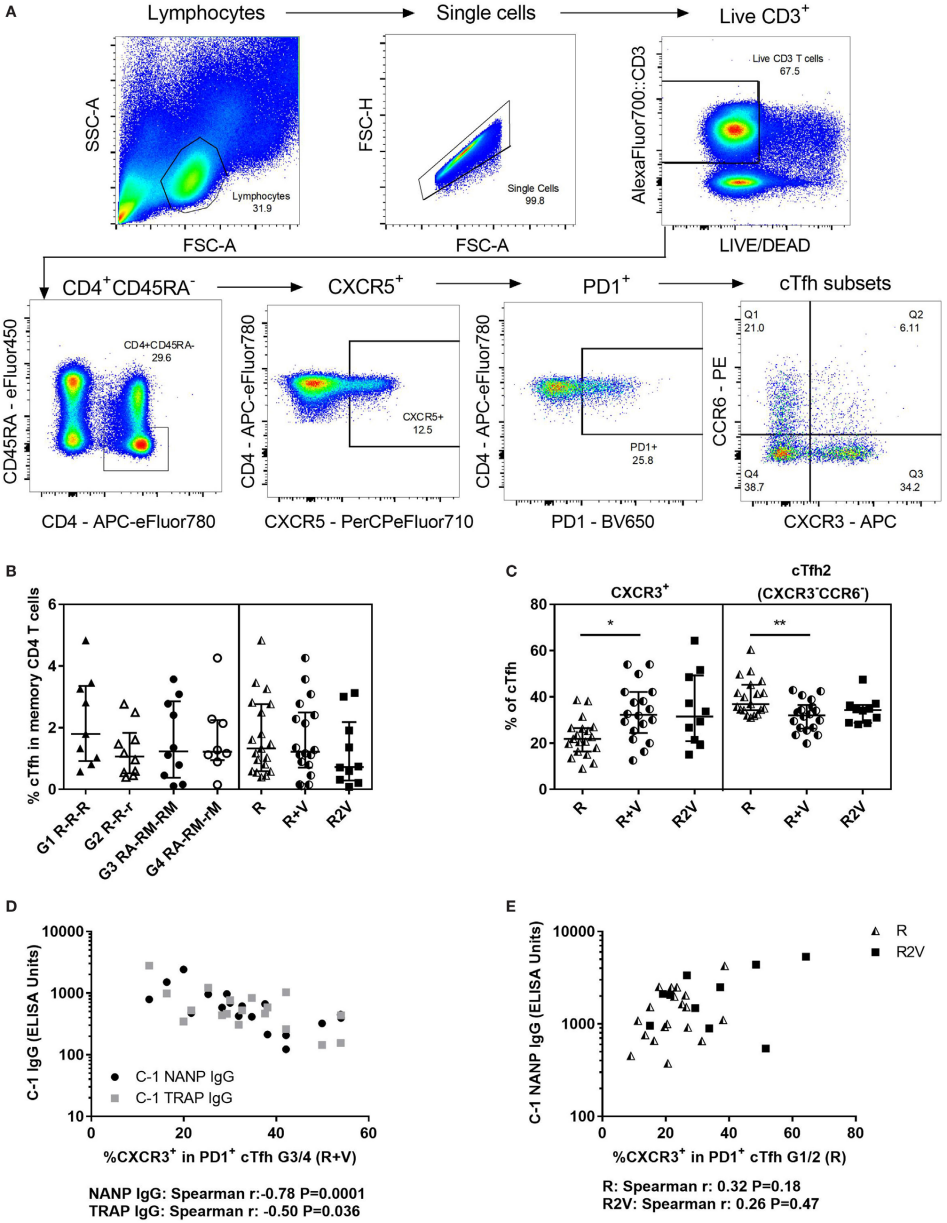
Total cTfhs. **(A)** Gating strategy for cTfh phenotyping using cell surface staining and flow cytometry. **(B)** Percentage of cTfh (PD1^+^CXCR5^+^) within memory CD4 T cells (CD45RA^−^) at C-1. **(C)** Subsets within cTfh (at C-1) defined by chemokine receptor expression: cTfh2 (CXCR3^−^CCR6^−^), or CXCR3^+^; including double-positive, dp (CXCR3^+^CCR6^+^), and cTfh1 (CXCR3^+^CCR6^−^). Kruskal–Wallis analyses; cTfh2 *P* = 0.009, CXCR3^+^ *P* = 0.01. **(D)** Relationship between percentage of CXCR3^+^ cTfh and antibody responses at C-1 in individuals who received RTS,S/AS01B co-administered with viral-vectored vaccines (G3/4). NANP IgG (Spearman *r*: −0.78, *P* = 0.0001), TRAP IgG (Spearman *r*: −0.50, *P* = 0.036). **(E)** Relationship between percentage of CXCR3^+^ cTfh and C-1 NANP IgG in individuals vaccinated with RTS,S/AS01B alone (R, G1/2, Spearman *r*: 0.32, *P* = 0.18) or RTS,S/AS01B and viral-vectored vaccines in a staggered regimen (R2V, Spearman *r*: 0.26, *P* = 0.47). Abbreviations: R G1/2, RTS,S/AS01B vaccinated; R + V G3/4, RTS,S/AS01B and viral-vectored vaccines co-administered; R2V, RTS,S/AS01B and viral-vectored vaccines at a 2-week stagger; A, ChAd63 ME-TRAP; M, MVA ME-TRAP; R, 50 µg third dose of RTS,S/AS01B; r, 10 µg third dose of RTS,S/AS01B; CHMI, controlled human malaria infection; C-1, day before CHMI; cTfh, circulating T follicular helper cell; TRAP, thrombospondin-related adhesive protein.

To determine whether cellular differences associated with the reduction in antibody responses in the co-administration regimen could be detected in the blood, cTfh were phenotyped at C-1 by surface staining and flow cytometry using a defined gating strategy (Figure 4A). Total cTfh were analyzed for all volunteers in the co-administration study (except 1 volunteer in G4 for which there were no cryopreserved cells remaining) and 10 volunteers in the staggered administration study with enough cryopreserved cells remaining for the experiment (Figure 4B). The proportion of cTfh (PD1^+^CXCR5^+^) within memory CD4^+^ T cells ranged from 0.1 to 4.8%, was comparable across groups and did not correlate with CSP- or TRAP-specific antibody responses. Subsets within cTfh were identified using CXCR3 and CCR6: cTfh17 (CXCR3^−^CCR6^+^), CXCR3^+^ [including double-positive (CXCR3^+^CCR6^+^) and cTfh1 (CXCR3^+^CCR6^−^)] and cTfh2 (CXCR3^−^CCR6^−^). Volunteers who received RTS,S/AS01B co-administered with viral-vectored vaccines had significantly higher frequencies of CXCR3^+^ cTfh and significantly lower frequencies of cTfh2 than those who received RTS,S/AS01B alone, while the staggered administration group had an intermediate phenotype which was not significantly different to either of the other regimens (Figure 4C). There were no significant differences in frequencies of any population between G1 R-R-R and G2 R-R-r or G3 RA-RM-RM and G4 RA-RM-rM (data not shown). In the co-administration regimen, the percentage of CXCR3^+^ cTfh was negatively correlated with antibody responses to both vaccines (Figure 4D, NANP Spearman *r*: −0.78, *P* = 0.0001, TRAP Spearman *r*: −0.50, *P* = 0.036). For groups that received RTS,S/AS01B alone, the frequency of CXCR3^+^ cTfh was lower, and there was no association with antibody responses (Figure 4E, Spearman *r*: 0.32, *P* = 0.18). Although in the staggered regimen the proportion of CXCR3^+^ cTfh was comparable to that in the co-administration regimen, they were not associated with a reduction in antibody responses in this regimen (Spearman *r*: 0.26, *P* = 0.47). The proportion of CXCR3^+^ in CXCR5^−^ memory CD4^+^ T cells was not associated with antibody responses in any regimen (data not shown).

### Co-Administration of Viral-Vectored Vaccines With RTS,S/AS01B Drives Th1-Biased Cytokine Responses Which Are Associated With the Increase in CXCR3^+^ cTfh and Reduction in Antibody Responses

**Figure 5:**
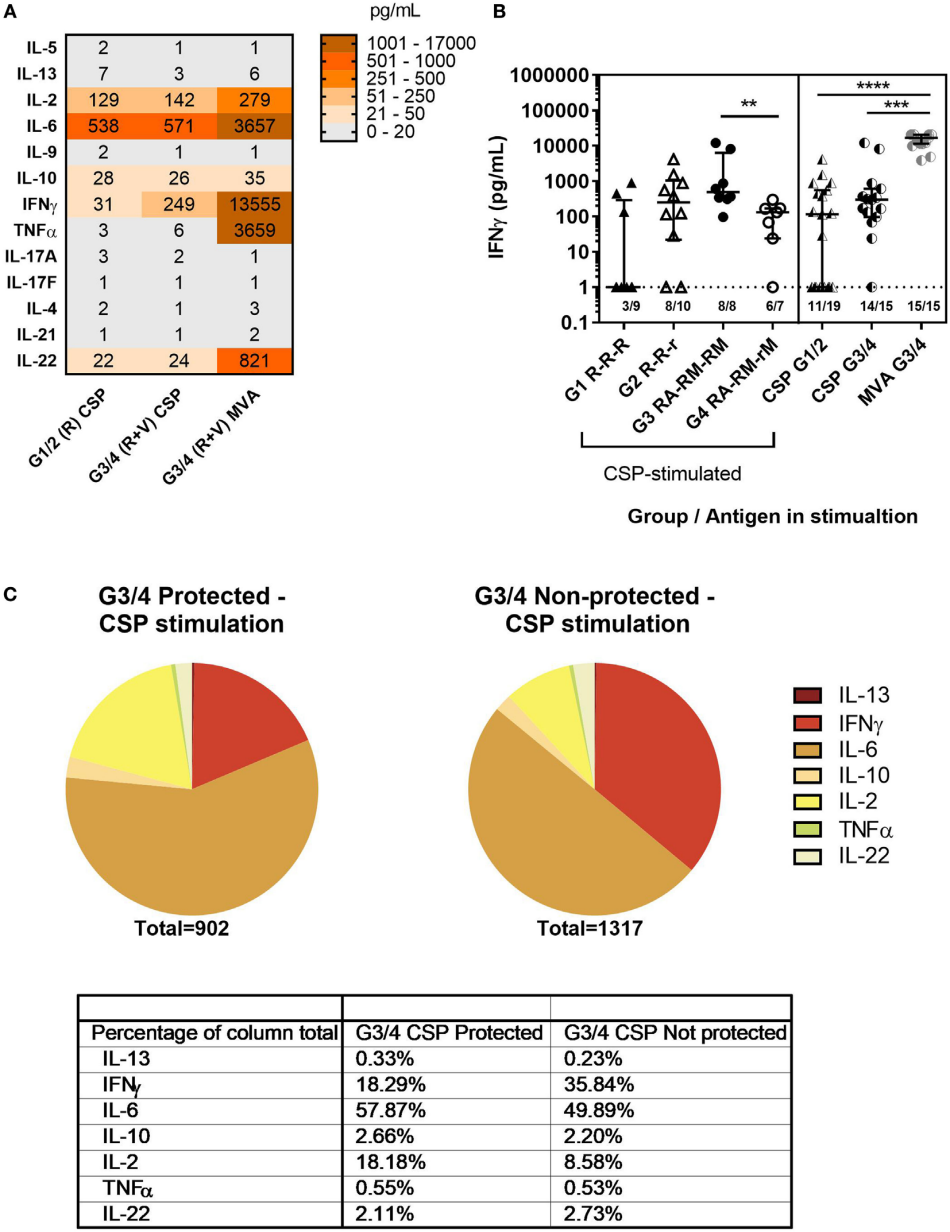
Cytokine responses to CSP and MVA. Cytokine responses measured in supernatant after stimulation of 1–2 × 10^6^ PBMC at C-1 with CSP or MVA. A multiplex cytokine bead assay (Legendplex, BioLegend) was used to measure a panel of T-helper cytokines. Responses were measured for 19 G1/2 samples stimulated with CSP and 15 G3/4 samples for which there were enough cells to run both CSP and MVA stimulations. **(A)** Heatmap of geomean cytokine concentrations in pg/mL in supernatant of PBMC from G1/2 stimulated with CSP and G3/4 stimulated with CSP or MVA. **(B)** Concentration of IFNγ in supernatant of PBMC stimulated with CSP or MVA, Kruskal–Wallis with Dunn’s multiple comparisons *P* < 0.0001. Mann–Whitney analysis between G3 RA-RM-RM and G4 RA-RM-rM *P* = 0.0037. Lower limit of detection for all cytokines in this assay was 1 pg/mL (indicated by the dashed line). **(C)** Proportions of cytokines produced in response to CSP stimulation (geomeans) in G3/4 (R + V) volunteers who were protected compared with those who were not. Table shows the level of each cytokine produced as a percentage of the total cytokine response to CSP in each group. Abbreviations: R G1/2, RTS,S/AS01B vaccinated; R + V G3/4, RTS,S/AS01B and viral-vectored vaccines co-administered; R2V, RTS,S/AS01B and viral-vectored vaccines at a 2-week stagger; A, ChAd63 ME-TRAP; M, MVA ME-TRAP; R, 50 µg third dose of RTS,S/AS01B; r, 10 µg third dose of RTS,S/AS01B; CHMI, controlled human malaria infection; C-1, day before CHMI; CSP, circumsporozoite protein; PBMCs, peripheral blood mononucleocytes; TRAP, thrombospondin-related adhesive protein.

Concentrations of a range of T-helper cytokines in the supernatant of C-1 PBMC from the co-administration study were measured using a cytometric bead array (LEGENDplex, BioLegend). PBMCs were stimulated with CSP (all groups) or MVA (G3&4) and concentrations of IL-5, IL-13, IL-2, IL-6, IL-9, IL-10, IFNγ, TNFα, IL-17A, IL17-F, IL-4, IL-21, and IL-22 were measured (Figure 5A). High concentrations of IL-2, IFNγ, TNFα, IL-6, and IL-22 were detected. The concentration of IFNγ was particularly high in the supernatant from MVA-stimulated PBMCs where all samples produced >3,000 pg/mL. In comparison, IFNγ responses were significantly lower after CSP stimulation, with no detectable IFNγ in 9/34 samples and less than 1,000 pg/mL in most where responses were detected. However, PBMCs from volunteers in the co-administration groups produced more IFNγ in response to CSP stimulation than those from volunteers who received RTS,S/AS01B alone (Figure 5B, median pg/mL + IQR, R CSP: 114 [1–348], R + V CSP: 311 [96–610], R + V MVA: 16,796 [11,409–20,462], Kruskal–Wallis *P* < 0.0001). In addition, IFNγ was a greater proportion of the cytokine response to CSP stimulation in PBMC from G3/4 (R + V) volunteers who were not sterilely protected after CHMI than those who were protected (Figure 5C).

**Figure 6:**
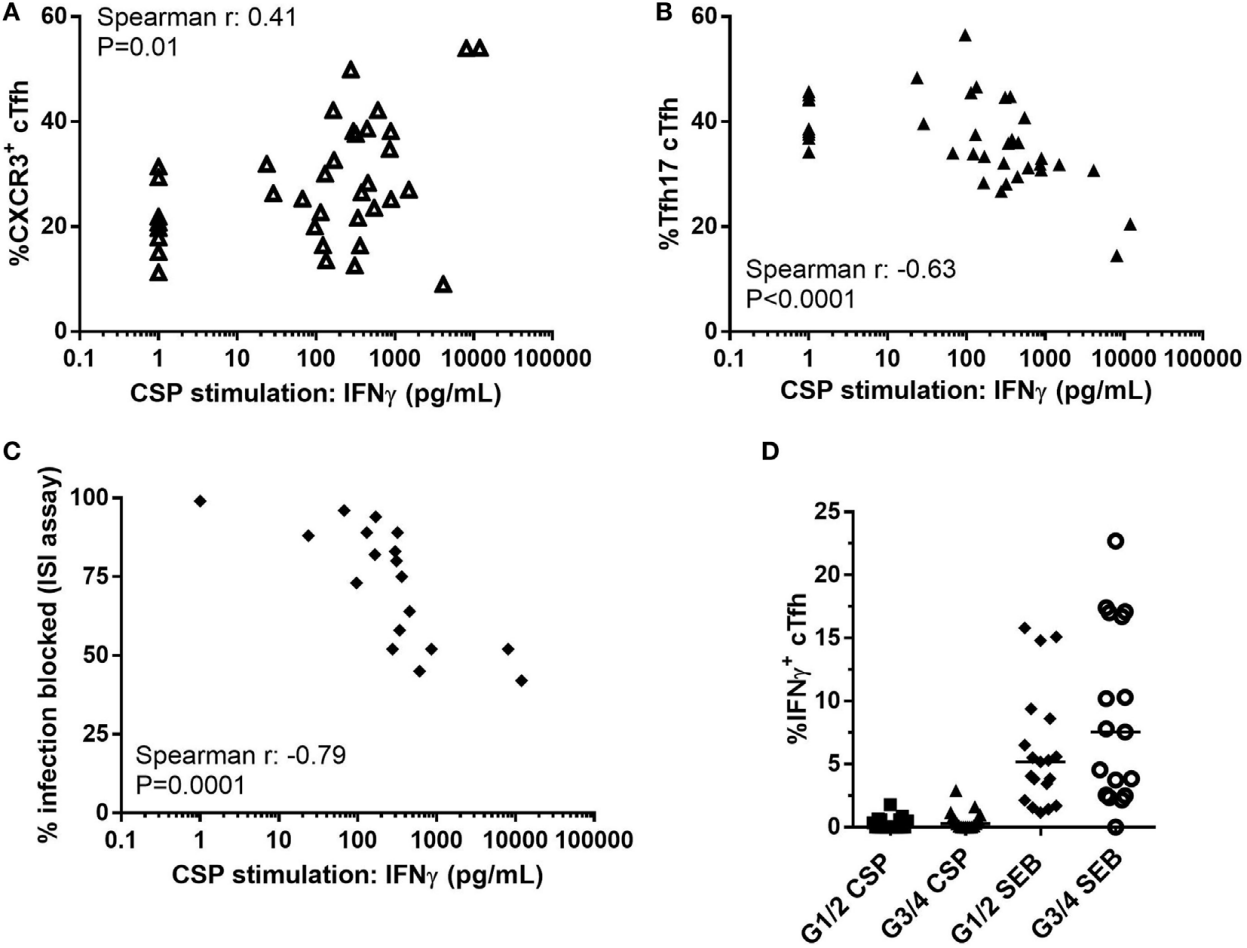
Association between Th1-biased cytokine responses and suppressed humoral immunity. Relationship between concentration of IFNγ in supernatant of PBMCs stimulated with CSP in co-administration trial (G1–4, R, and R + V combined) and: **(A)** Proportion of CXCR3^+^ cTfh (Spearman *r*: 0.41, *P* = 0.01); **(B)** Proportion of cTfh17 (CXCR3^+^CCR6^−^) within cTfh (Spearman *r*: -0.63, *P* < 0.0001); **(C)** Percentage of infection blocked in the ISI assay (Spearman *r*: -0.79, *P* = 0.0001). **(D)** Proportion of IFNγ^+^ cTfh after stimulation with CSP or SEB, Mann–Whitney analyses between G1/2 (R) and G3/4 (R + V) *P* = 0.14 and *P* = 0.26 for CSP and SEB stimulations, respectively. Abbreviations: R G1/2, RTS,S/AS01B vaccinated; R + V G3/4, RTS,S/AS01B and viral-vectored vaccines co-administered; R2V, RTS,S/AS01B and viral-vectored vaccines at a 2-week stagger; A, ChAd63 ME-TRAP; M, MVA ME-TRAP; R, 50 µg third dose of RTS,S/AS01B; r, 10 µg third dose of RTS,S/AS01B; CHMI, controlled human malaria infection; C-1, day before CHMI; CSP, circumsporozoite protein; cTfh, circulating T follicular helper cell; ISI, inhibition of sporozoite invasion; PBMCs, peripheral blood mononucleocytes; TRAP, thrombospondin-related adhesive protein; SEB, *Staphylococcus* enterotoxin B.

The concentration of IFNγ in the CSP supernatant was positively associated with the proportion of CXCR3^+^ cTfh (Figure 6A, Spearman *r*: 0.41, *P* = 0.01) and negatively with the proportion of cTfh17 within cTfh (Figure 6B, Spearman *r*: −0.63, *P* < 0.0001). In addition, there was a negative association between the concentration of IFNγ in the CSP supernatant and the ability of antibody to block sporozoite entry into hepatocytes (Figure 6C, Spearman *r*: −0.79, *P* = 0.0001). Analysis of IFNγ production by ICS of cTfh after stimulation with CSP or the superantigen SEB showed very low frequencies of these cells expressing IFNγ [Figure 6D, less than 2% in G1/2 (R) and less than 3% in G3/4 (R + V) after CSP stimulation].

## Discussion

Viral-vectored vaccines ChAd63 ME-TRAP and MVA ME-TRAP given with RTS,S/AS01B in a staggered regimen induced high titers of antibodies against sporozoites and potent T cell responses against infected liver cells (18). This regimen required five separate vaccinations and would likely be impractical and uneconomical for deployment in malaria-endemic regions. One way to overcome this obstacle would be to co-administer the RTS,S/AS01B and viral-vectored vaccines. Co-administration of vaccines can lead to interactions between the immune responses, which may be beneficial, enhancing the response as seen after co-administration of the live and attenuated polio vaccines or may result in a reduction of immunogenicity as is the case for multivalent inactivated or live viral vaccines (30, 31).

We examined the immune responses induced by both vaccines when co-administered to assess the extent of the immunological interaction between RTS,S/AS01B and viral-vectored vaccines. An *in vitro* assay measuring the ability of vaccine-induced antibody to block sporozoite invasion of hepatocytes was used to assess the functional quality of antibody. Infection-blocking ability was associated with protection. However, some volunteers who received viral-vectored vaccines were protected despite having antibodies with only low levels of infection-blocking activity. These volunteers had some of the highest TRAP-specific T cell responses, suggesting that cellular responses may provide protection by killing infected hepatocytes in volunteers who do not produce sufficient anti-NANP titers to block sporozoite entry. This demonstrates the potential of a multistage targeting regimen to provide high-level efficacy if each vaccine can be given without interfering with the immunogenicity of the other. However, co-administration of viral-vectored vaccines with RTS,S/AS01B in this study induced a strong Th1 cytokine response and increased proportions of CXCR3^+^ cTfh, which were associated with reduced antibody quantity and quality and lower efficacy in these groups.

In addition, we observed qualitative differences in RTS,S-induced antibody responses when a reduced third (1/5th, 10 µg) dose was given compared with three standard doses (50 µg). Although NANP IgG titers were comparable, administration of a reduced third dose of RTS,S/AS01B-induced antibodies that were capable of blocking a significantly higher level of sporozoite infection *in vitro*—a measure which was associated with protection from CHMI. Previously, a reduced third dose of RTS,S/AS01B was shown to provide higher levels of protection (14, 19) and in a more recent study, a fractional third dose boost induced antibody with increased somatic hypermutation and higher avidity (19). However, it was unclear whether this effect was due to the delayed boost (0, 1, and 7-month regimen) or the fractional dose. Our study is the first to demonstrate a functional difference in the antibodies induced by the reduced third dose regimen that is associated with protection. However, it is unclear whether the quality of the antibody response is enhanced by a reduced third dose in particular or whether this could be achieved with a reduction of all three doses, which would also have economical and practical advantages. Lower vaccine doses decrease the availability of antigen and therefore could result in greater affinity maturation through increased competition between B cells for T cell help and preferential expansion of B cell clones with the highest affinity B cell receptors (32, 33). Lower doses of antigen at priming also preferentially drive the induction of memory, while higher antigen doses drive differentiation of plasma cells (34). The preferential induction of memory by reducing the priming dose could enhance responses to the subsequent vaccinations and also potentially generate more durable protection. This suggests that the dosing regimen should also be carefully tested to ensure the optimal type of immune response is achieved.

Previous studies have demonstrated that cTfh may be useful biomarkers for GC responses in the absence of lymphoid tissue (35). However, cTfh are a heterogeneous population composed of a number of different subsets, some of which appear to more closely resemble *bona fide* GC Tfh than others (22, 36). The proportions of these subsets have been associated with different diseases: increases in cTfh2/cTfh17 subsets are associated with the production of autoantibodies and disease severity in various autoimmune diseases (22, 37, 38), the development of allergy (39, 40) and the production of broadly neutralizing antibody in HIV^+^ individuals (26, 41). By contrast, increases in CXCR3^+^ cTfh have been implicated in the poor development of humoral immunity against malaria (42, 43) and are proportionally increased in patients with primary immunodeficiencies (44). In addition, a study that observed CXCR3^+^ cTfh to positively correlate with antibody responses after influenza vaccination also showed that CXCR3^+^ Tfh that were localized to tonsillar GCs, expressed Fas-L, secreted IFNγ, lacked CD154 expression, and suppressed the activity of GC B cells. Therefore, although this subset was correlated with antibody responses, they were not optimal for their induction (45). In our study, co-administration of RTS,S/AS01B with viral-vectored vaccines led to an increased frequency of CXCR3^+^ total cTfh compared with RTS,S/AS01B administered alone, and this phenotype was associated with the observed reduction in antibody quantity and quality. Although the observed association was for total cTfh, it would be useful to profile the antigen-specificity of these cells to determine if the increase in CXCR3^+^ cTfh was due to the induction of cTfh specific for the vector or whether this was a change in the phenotype of the CSP-specific cTfh. There are several methods used to look at antigen-specific cTfh, including cytokine production or CD154 expression after overnight antigen stimulation or the use of antigen-induced markers (45–48). Unfortunately, we found that staining for the chemokine receptors CXCR3 and CCR6 could not reliably be incorporated into these assays (unpublished data). Therefore, we were unable to determine the specificity of the CXCR3^+^ cTfh.

Circulating Tfh have been shown to produce cytokines, with CXCR3^+^ cTfh in particular producing IFNγ (49). However, the association we saw between IFNγ in the supernatant and CXCR3^+^ cTfh was not likely due to production of IFNγ by the CXCR3^+^ cTfh themselves as only very low percentages of cTfh were observed to produce IFNγ after CSP or SEB stimulation. This suggests that exogenous sources of IFNγ are associated with the polarization of cTfh toward a CXCR3^+^ phenotype, although vector-specific CXCR3^+^ cTfh could be a source IFNγ in G3/4 volunteers. MVA has previously been shown to drive a strong IFNγ response, particularly in CD8^+^ T cells and IFNγ enhances CXCR3 expression on T cells through STAT1 signaling (50–53). It is perhaps therefore unsurprising that MVA induced a CXCR3-skewed cTfh response. However, the impact of this skew on the antibody responses was less predictable, given that CXCR3^+^ cTfh have in some contexts been positively associated with antibody responses (45, 54) while in other studies they have been associated with suboptimal GC responses and poor humoral immunity (42, 55). In our study when the two different vaccine platforms were co-administered, the IFNγ-dominated cytokine responses driven by viral-vectored vaccines were associated with an increase in CXCR3^+^ cTfh and a reduction in humoral immunity and protective efficacy. However, if given 2 weeks after RTS,S/AS01B the extent of this CXCR3^+^ cTfh skew, although only slightly reduced, is no longer associated with a reduction in humoral immunogenicity.

IFNγ induces the production of chemokines CXCL9 (MIG, monokine induced by gamma-interferon), CXCL10 (IP-10, interferon-induced protein of 10 kDa), and CXCL11 (I-TAC, interferon inducible T cell alpha chemoattractant), which all bind CXCR3. This chemokine system mediates the migration of Th1 CD4^+^ T cells and cytotoxic T lymphocytes to sites of Th1 inflammation in the periphery (56). MVA has been shown to induce high systemic levels of IP-10 (57). The systemic induction of CXCR3 ligands, in combination with the CXCR3^+^ cTfh polarization, may result in the reduction of antibody responses by causing an egress of these cTfh from the draining lymph node, preventing them from providing help to B cells in the GC response. A staggered regimen may reduce this effect by allowing time for the RTS,S/AS01B-induced GC response to occur before MVA-induced inflammation begins. Alternative strategies to reduce or avoid the observed immune interference without increasing the number of clinic visits could be to reduce the dose of MVA or to exclude the additional MVA vaccination at week 4, which was included for practical reasons to simplify the vaccination protocol and is not required to induce potent T cell responses (17, 18).

Producing an effective vaccine against malaria will likely depend on a combination of vaccines targeting multiple stages of the parasite lifecycle. In resource-poor settings, mixing, or co-administering, the vaccines will be necessary to reduce the number of clinic visits required, particularly in infants to fit in with the established Expanded Program on Immunisation vaccine schedule. However, the effects of co-administration on immunogenicity and protective efficacy of each vaccine must be carefully examined. Ideally, a combination regimen could be designed to elicit antibody and T cell responses with an additive protective effect and without the immune interference observed here. Examining the cellular mechanisms underlying these differences in antibody responses will be critical for determining how effective, long-lived antibody responses can be induced by vaccination and for informing rational design of vaccine regimens.

## Ethics Statement

All volunteers gave written informed consent prior to participation, and the studies were conducted according to the principles of the Declaration of Helsinki and in accordance with Good Clinical Practice. The trials were registered with ClinicalTrials.gov (Ref: NCT01883609 and NCT02252640). The study protocols were approved by the UK National Research Ethics Service, Committee South Central—Oxford A (Refs: 13/SC/0208 and 14/SC/0227), the Western Institution Review Board (Ref: 20130698) and the UK Medicines and Healthcare products Regulatory Agency (Refs: 21584/0317/001-0001 and 21584/0333/001-0001). The Local Safety Committee provided safety oversight for both trials and GCP compliance was monitored by the Clinical Trials and Research Governance Team (CTRG) of the University of Oxford.

## Author Contributions

GB designed laboratory study, interpreted data, and wrote the manuscript. GB and AG acquired and analyzed data. DM, RB, AH, and KE conceived clinical studies and provided project oversight. TR, NV, AH, and KE designed clinical studies. TR and NV conducted clinical work. All the authors revised the manuscript.

## Disclaimer

The views expressed are those of the author(s) and not necessarily those of the PATH Malaria Vaccine Initiative, the United Kingdom National Health Service, the United Kingdom NIHR, or the Department of Health.

## Conflict of Interest Statement

AH is a named inventor on patent applications covering malaria vectored vaccines and immunization regimens. DM and RB are employees of GSK, which is developing vaccines for malaria and other diseases. All other authors report no potential conflicts.

## Supplementary Material

Figure S1 Relationship between NANP-specific isotypes/subclasses and inhibition of sporozoite invasion (ISI). Associations between NANP-specific isotypes and subclass titers at C-1 and ISI **(A)** IgG1, **(B)** IgG2, **(C)** IgG3, **(D)** IgM, and **(E)** IgA.

## Abbreviations

ChAd, chimpanzee adenovirus; CHMI, controlled human malaria infection; CSP, circumsporozoite protein; cTfh, circulating T follicular helper cell; GC, germinal center; ISI, inhibition of sporozoite invasion; ME, multi-epitope; MVA, modified vaccinia virus Ankara; PBMCs, peripheral blood mononucleocytes; pfu, plaque-forming units; TRAP, thrombospondin-related adhesive protein; VE, vaccine efficacy; VP, viral particles.
