# Supplementary figures and images for: CXCR3+ T Follicular Helper Cells Induced by Co-Administration of RTS,S/AS01B and Viral-Vectored Vaccines Are Associated With Reduced Immunogenicity and Efficacy Against Malaria
Source: Front Immunol. 2018 Jul 25;9:1660. doi: 10.3389/fimmu.2018.01660 (PMC6068239; doi:10.3389/fimmu.2018.01660)

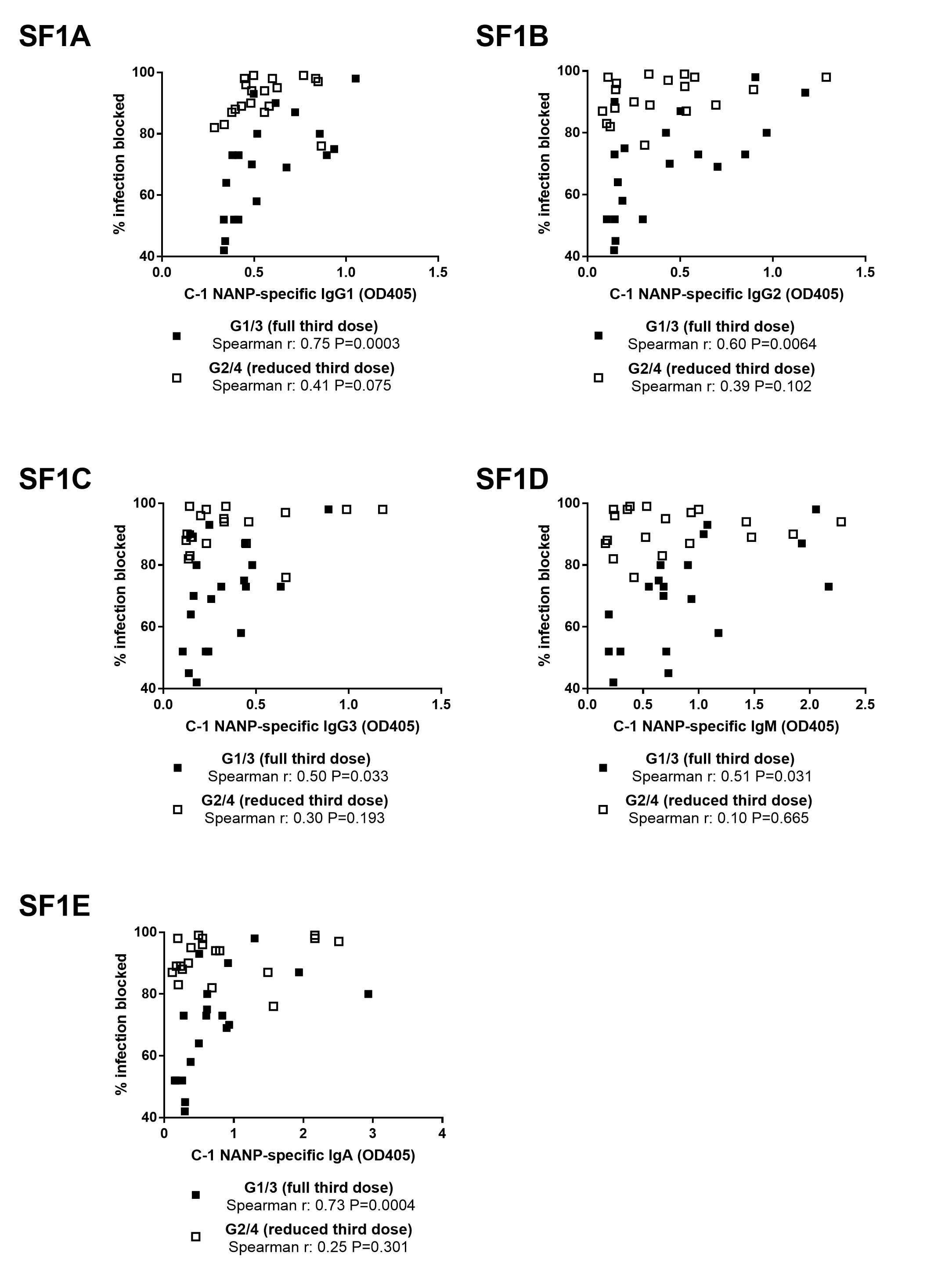

Supplement: Figure S1 — Relationship between NANP-specific isotypes/subclasses and inhibition of sporozoite invasion (ISI). Associations between NANP-specific isotypes and subclass titers at C-1 and ISI (A) IgG1, (B) IgG2, (C) IgG3, (D) IgM, and (E) IgA. [file Image_1.JPEG]
